# Issue of Veracity between Dr. Robert Battey and Mr. Lawson Tait

**Published:** 1886-08

**Authors:** Lawson Tait

**Affiliations:** Birmingham, England


### ISSUE OF VERACITY BETWEEN DR. ROBERT BATTEY AND MR. LAWSON TAIT.

MR. LAWSON TAIT CRITICISES THE JOURNALS' COMMENTS ON HIS RECENT LETTERS TO THE NEWS AND EXPLAINS THE SEEMING INACCURACIES IN HIS REPORTS, ETC.

*Editors of the Atlanta Medical and Surgical Journal:*

SIRS—You have reprinted from the *Medical News*, of May 15, a letter from Dr. Robert Battey, and you say that "that letter is calculated to impress upon American readers certain misgivings as to the trustworthiness of the reports made by Mr. Lawson Tait in regard to his gynecological work. If an operator varies his statements of results in given cases to suit his ulterior views, or to make the issue presentable by the experience of others, we have no safe guide to a discussion of any mortality questioned in the published data of such a surgeon, and Mr. Tait should learn that facts are stubborn things."

I venture to think, sirs, that this is an extremely unfair comment to make upon any one-sided statement until the other side of the question could be heard, and it is singularly out of agreement with the general courtesy displayed by your countrymen. I trust that in a spirit of fair play you will reproduce my answer to Dr. Battey's letter, which I have just forwarded to the editor of the *Medical News* in Philadelphia, and a copy of which I now enclose. I merely desire to point out with particular emphasis, in answering your insinuation, that the original mistake concerning the case in question was made against myself. If, on the contrary, I had published the case as a successful one, and ultimately had to admit that it had resulted in a fatal issue, then I think perhaps your insinuation might have had some grounds. But seeing that the error is one in the other direction, I think it might have been pardoned, and it was fully explained in Dr. Battey's hearing. The mistake was that I published a case first as a fatal one, and then, finding that the patient was still alive and that the record had been confused with another patient, I had to confess my mistake and admit that the patient was alive and quite cured.

I am, etc., LAWSON TAIT.

Birmingham, June 16, 1886.

